# A glow of HLA typing in organ transplantation

**DOI:** 10.1186/2001-1326-2-6

**Published:** 2013-02-23

**Authors:** Batool Mutar Mahdi

**Affiliations:** 1 Department of Microbiology, Director of HLA Typing research Unit, Al-Kindy College of Medicine, Baghdad University, AL-Nahda Square, Baghdad, Iraq

**Keywords:** Transplantation, Rejection, Major histocompatibility

## Abstract

The transplant of organs and tissues is one of the greatest curative achievements of this century. In organ transplantation, the adaptive immunity is considered the main response exerted to the transplanted tissue, since the main goal of the immune response is the MHC (major histocompatibility complex) molecules expressed on the surface of donor cells. Cell surface molecules that induce an antigenic stimulus cause the rejection immune response to grafted tissue or organ. A wide variety of transplantation antigens have been described, including the major histocompatibility molecules, minor histocompatibility antigens, ABO blood group antigens and endothelial cell antigens. The sensitization to MHC antigens may be caused by transfusions, pregnancy, or failed previous grafts leading to development of anti-human leukocyte antigen (HLA) antibodies that are important factor responsible for graft rejection in solid organ transplantation and play a role in post-transfusion complication Anti-HLA Abs may be present in healthy individuals. Methods for HLA typing are described, including serological methods, molecular techniques of sequence-specific priming (SSP), sequence-specific oligonucleotide probing (SSOP), Sequence based typing (SBT) and reference strand-based conformation analysis (RSCA) method. Problems with organ transplantation are reservoir of organs and immune suppressive treatments that used to decrease rate of rejection with less side effect and complications.

## Introduction

The HLA system includes a complex array of genes located on chromosome number 6 and their molecular products that are involved in immune regulation and cellular differentiation. Human leukocyte antigen (HLA) molecules are expressed on almost all nucleated cells, and they are the major molecules that initiate graft rejection. There are three classical loci at HLA class I: HLA-A, -B, and -Cw, and five loci at class II: HLA-DR, -DQ, -DP, -DM, and -DO. The system is highly polymorphic [1]. The contribution of the allelic diversity of class I and II genes to immune recognition and alloreactivity can be analyzed by serological methods and molecular methods at the DNA level by different methods like sequence specific primer (SSP) and oligotyping with locus- and allele-specific oligonucleotide probes (SSOP) [2]. HLA class I and II matching is important in organ transplantation [3] especially in kidney and bone marrow transplantation. In heart and lung transplantation, HLA match at the DR locus is important but there is some difficulties like ischemic times, availability of donors and clinical need of recipients. Corneal grafts are not usually influenced by HLA matching, unless being transplanted into a vascularized bed [4]. Transplantation of foreign tissue induces both humoral and cellular immune responses in the recipient, which leads to graft rejection or, for bone marrow transplantation, graft versus host disease (GVHD).

### Classification

MHC class I proteins form a functional receptor on most nucleated cells of the body. There are 3 major and 3 minor MHC class I genes in HLA: HLA-A,HLA-B and HLA-C; minor genes are HLA-E, HLA-F and HLA-G [5]. β_2_- microglobulin binds with major and minor gene subunits to produce a heterodimer. There are 3 major and 2 minor MHC class II proteins encoded by the HLA. The genes of the class II combine to form heterodimeric (αβ) protein receptors that are typically expressed on the surface of antigen-presenting cells. Major MHC class II HLA-DP: α-chain encoded by HLA-DPA1 locus and β-chain encoded by HLA-DPB1 locus. HLA-DQ: α-chain encoded by HLA-DQA1 locus and β-chain encoded by HLA-DQB1 locus. The last one is HLA-DR: α-chain encoded by HLA-DRA locus and four β-chains (only 3 possible per person), encoded by HLA-DRB1, DRB3, DRB4, and DRB5 loci. The other MHC class II proteins, DM and DO, are used in the internal processing of antigens, loading the antigenic peptides generated from pathogens onto the HLA molecules of antigen-presenting cell [6].

### Nomenclature

Two systems of nomenclature are applied to HLA. The first system is based on serological recognition. In this system, antigens were eventually assigned letters and numbers (e.g., HLA-B51 or, shortened, B51). Modern HLA nomenclature are begin with HLA- and the locus name, then * and even number of digits specifying the allele. The first two digits specify a group of alleles. Older typing methodologies often could not be completely distinguish alleles and so stopped at this level. The third through fourth digits specify a synonymous allele. Digits five through six denote any synonymous mutations within the coding frame of the gene. The seventh and eighth digits distinguish mutations outside the coding region. Letters such as L, N, Q, or S may follow an allele's designation to specify an expression level or other non-genomic data known about it. Thus, a completely described allele may be up to nine digits long, not including the HLA-prefix and locus notation, (e.g., HLA- A*24:02:01 N N=Null) to designate a specific allele at a given HLA locus. Every two years, a nomenclature is put forth to aid researchers in interpreting serotypes to alleles [7].

### Variability

Since the biologic function of the HLA molecules is presenting endogenous and exogenous antigens, they manifested high structural polymorphism. Until 2010, 2558 HLA class I and II alleles have been recognized [8]. HLA presents the highest degree of polymorphism of all human genetic systems. One mechanism of this polymorphism is due to gene conversion between variable alleles and loci within each HLA gene [9]. The number of HLA allele's increases at a regular rate, about 15% of the alleles in the high-expression loci (A, B, C, and DRB1) and 30% in the low-expression loci (DRB3/4/5, DQB1, DPB1) are truly relevant in clinical typing. The number of alleles will keep growing. The number of variant alleles at class I and class II loci according to the IMGT-HLA database, last updated June 2012: as shown in Tables 1 and 2[10].

**Table 1:** The number of variant alleles at class I loci

**MHC class I**	
**Locus**	**No.**
**Major Antigens**	
**HLA A**	1,884
**HLA B**	2,490
**HLA C**	1,384
Minor Antigens	
HLA E	11
HLA F	22
HLA G	49

**Table 2:** The number of variant alleles at class II loci

**MHC class II**			
**HLA**	-**A1**	-**B1**	**-B3 to -B5 **^**1**^
**Locus**	**No.**	**No.**	**No.**
**DM-**	7	13	
**DO-**	12	13	
**DP-**	34	155	
**DQ-**	47	165	
**DR-**	7	1,094	92

^1^DRB3, DRB4, DRB5 have variable presence in humans.

### HLA typing and its influence on organ transplantation

The selection of the optimal donor is based on high-resolution HLA typing. The MHC (Major Histocompatibility Complex) contains more than 200 genes which are situated on the short arm of chromosome 6 at 6p21.3 The role of HLA molecules is to present peptides to T cells (both CD4 and CD8 T cells), enabling them to recognize and eliminate “foreign” particles and also to prevent the recognition of “self” as foreign. HLA mismatches may occur at antigenic or allelic level; the first are characterized by amino acid substitutions in both peptide binding and T-cell recognition regions, whereas the latter are characterized by amino-acid substitution in the peptide binding regions only [11]. When a human transplant is performed, HLA (human leukocyte antigens) molecules from a donor are recognized by the recipient's immune system by direct and indirect methods of allorecognition triggering an alloimmune response. Matching of donor and recipient for MHC antigens showed a significant positive effect on graft acceptance [12]. In organ transplantation, the adaptive immunity is the main response exerted to the transplanted tissue, since the main target of the immune response is the MHC molecules expressed on the surface of donor cells. T-cell activation leads to the production of cytokines and chemokines which in turn may recruit components of the innate immunity like NK cells or macrophages and complement [13]. In addition to that, defensins and cathelicidin have chemoattractant properties on T lymphocytes [14].

In transplantation immunology, the major impact in graft loss comes from the effects of HLA-B and -DR antigens [15]. The effects of HLA-DR mismatches are the most important in the first 6 months after transplantation, the HLA-B effect emerges in the first 2 years, and HLA-A mismatches have a deleterious effect on long-term graft survival [16].

### Techniques of tissue typing

Previously, HLA typing was done by two methods: serologic method using antiserum and mixed lymphocyte culture (MLC). After that a more precise DNA-based HLA typing methods using molecular techniques, such assequence-;specific oligonucleotide probe hybridization (SSOP), sequence-specific primer amplification (SSP), sequencing-based typing (SBT), and reference strand-based conformation analysis (RSCA), have been developed and are frequently used [17]. In 2013, a new project of the 16IHIW demonstrated the potential benefits of next-generation sequencing (NGS) in the HLA laboratory. NGS may resolve the issue through the combination of clonal amplification, which provides phase information, and the ability to sequence larger regions of genes, including introns, without the additional effort or cost associated with current methods [18]. Another simplified method using short tandem repeat (STR) genotyping provided additional information allowing determination of the extent of HLA identity in families where HLA haplotype inheritance was ambiguous, due to extensive homozygosity or shared parental haplotypes. The HLA STR assay is a reliable and rapid test that used inexpensively screen potential sibling donors for HLA identity [19].

First method is serological assay, can be made by antibody-dependent cell-mediated cytotoxicity (ADCC), or complement-dependent cytotoxicity (CDC). |In ADCC, the cytotoxic destruction of antibody-coated target cells by host cells is triggered when an antibody bound to the surface of a cell interacts with Fc receptors on NK cells or macrophages. Serologic method was also used in screening for HLA antibodies in the recipient. These antibodies are important because they are reactive with lymphocytes of a prospective donor (cross matching) [20,21].

Concerning the cellular assay, this is a mixed lymphocyte culture and used to determine the HLA class II types. The cellular assay is more sensitive in detecting HLA differences than serotyping. This is because minor differences unrecognized by alloantisera can stimulate T cells. This typing is designated as Dw types [22,23].

Tissue typing by molecular method, utilizing the sequence-specific oligonucleotide (SSO) and sequence-specific primer (SSP) technologies has been in routine use in many tissue-typing laboratories worldwide for more than 20 years since the development of the polymerase chain reaction. Both methods are very useful for clinical and research purposes and can provide generic (low resolution) to allelic (high resolution) typing results [24]. DNA based method had more sensitivity, accuracy and resolving power than serologic typing methods [25]. Sequencing-based typing (SBT) is a high-resolution method for the identification of HLA polymorphisms. The majority of HLA Class I alleles can be discriminated by their exon 2 and 3 sequence, and for Class II alleles, exon 2 is generally sufficient. There are polymorphic positions in other exons, which may require additional sequencing to exclude certain alleles with differences outside exon 2 and 3, depending on the clinical requirement and relevant accreditation guidelines. Examination of all nucleotides, both at conserved and polymorphic positions enables the direct identification of new alleles, which may not be possible with techniques such as SSP and SSO typing [26].

Regarding antibodies screening was done to avoid hyperacute rejection, it is very important to identify recipient anti-HLA antibodies to antigens expressed on donor with blood cells [27]. The pioneer method to detect such antibodies is complement-dependent cytotoxicity (CDC), in the mid 1990s, it has been gradually replaced by more-sensitive solid-phase immunoassays (SPI) such as the enzyme-linked immunosorbent assay and the bead-based technology (i.e., flow cytometry: Flow PRA and Flow Analyzer-Luminex). These assays used microparticles coated with purified HLA molecules. Hence, the era of solid-phase microparticle technology for HLA antibody detection was born permitting the sensitive and specific detection of HLA antibody. A comprehensive list of recommendations is provided covering the technical and pretransplantation and posttransplantation monitoring of HLA antibodies in solid organ transplantation like SPI must be used for the detection of pretransplantation HLA antibodies in solid organ transplant recipients and in particular the use of the single-antigen bead assay to detect antibodies to HLA loci such as Cw, DQA, DPA, and DPB, which are not readily detected by other methods. The use of SPI for antibody detection should be supplemented with cell-based assays to examine the correlations between the two types of assays and to establish the likelihood of a positive crossmatch (XM). There must be an awareness of the technical factors that can influence the results and their clinical interpretation when using the Luminex bead technology, such as variation in antigen density and the presence of denatured antigen on the beads. The recommendations are intended to provide state-of-the-art guidance in the use and clinical application of recently developed methods for HLA antibody detection when used in conjunction with traditional methods [28].

It is hoped that these technologies will ultimately lead to the identification of parameters that best correlate with and/or predict transplant outcomes [29]. However, the new techniques have been associated with decreased specificity, and some non-HLA antigens with no clinical relevance have been able to give a positive cross match [30]. These “false-positive” antibody results have a consequence of decreasing a chance of the patient to receive an organ [31]. Susal and Opelz (2012) [32] done a collaborative transplant study; they summarized that cold ischemia up to 18 hours was found not to be detrimental for graft outcome, and short cold ischemia did not eliminate the effect of HLA matching [33] and HLA-DR mismatches were a significant risk factor for posttransplant non-Hodgkin lymphoma and hip fracture [34,35]. Meier-Kriesche et al. 2009 [36], who examined some 16,000 renal transplant candidates in the USA that were re-listed after loss of a primary kidney transplant, found that with increasing numbers of HLA mismatches of the first transplant, there was a significant increase in panel reactive antibody. They also observed a pronounced influence of HLA matching on graft outcome in patients with pretransplant HLA antibodies or high serum levels of the T-cell activation marker sCD30 [37]. Although graft survival rates improved greatly now a days, sensitization of the recipient at the B-cell level and development of IgG antibodies still represents a major problem in kidney transplantation that detected in 17% of patients after first transplant by ELISA screening assay and using the more sensitive Luminex technology, HLA antibodies of the IgG, IgM, or IgA isotypes were detected in almost all patients waiting for transplant [38]. Other types of antibodies that directed against non-HLA antigens are important like against graft endothelium [39], MICA [40] and Angiotensin type 1 receptor [41]. To reduce such presumed undetected antibody activity below clinically relevant threshold levels peritransplant apheresis was indicated [42]. Heinold et al 2013 [43] reported that HLA compatibility significantly influences the outcome of kidney transplants in sensitized retransplant recipients even at the allele level. In addition to that, the interaction between recipient HLA ligand and donor KIR had a significant impact on the outcome of patients receiving HLA matched sibling [44].

### What are the future prospects for transplantation?

Organ transplants have helped thousands of people. The main obstacles are reservoir of organs, so we need to increase the number of available organs and find more precise methods of immunosuppression in order to prevent rejection without the dangerous side effects of infection and cancer.

## Conclusions

Expansion the field of organ and tissue transplantation has accelerated amazingly, since the human major histocompatibility complex (MHC) was discovered in 1967. Transplantation immunology is a very complex process and the numbers of alleles were increased remarkably. The development of techniques that used in tissue typing laboratory was also developed than previous. All together worked in order to get graft acceptance for long period of time.

## Review

Considering the role of HLA matching in transplant outcome. In current years, serological methods have been replaced with DNA based typing methods. The main objective of this study is reviewing the important effect of HLA typing, polymorphism, classification, different techniques used and detecting antibodies against transplanted organ by diverse methods.

## Competing interest

The author declare that he has no competing interests.
